# Collision Prevention for Duty-Cycle Receiver-Initiation MAC Protocol via Multiple Access Reservation (MAR-RiMAC)

**DOI:** 10.3390/s21010127

**Published:** 2020-12-28

**Authors:** Omer Gurewitz, Oren Zaharia

**Affiliations:** School of Electrical and Computer Engineering, Ben-Gurion University of the Negev, Beer-Sheva, IL 8410501, USA; orenzah@post.bgu.ac.il

**Keywords:** wireless sensor networks (WSNs), IoT, medium access control (MAC), asynchronous duty cycling, receiver-initiated

## Abstract

The prevalence of the Internet of Things (IoT) paradigm in more and more applications associated with our daily lives has induced a dense network in which numerous wireless devices, many of which have limited capabilities (e.g., power, memory, computation), need to communicate with the internet. One of the main bottlenecks of this setup is the wireless channel. Numerous medium access control (MAC) protocols have been devised to coordinate between devices that share the wireless channel. One prominent approach that is highly suitable for IoT and wireless sensor networks (WSNs), which rely on duty cycling, is the receiver-initiated approach, in which, rather than the transmitter, the receiver initiates the transaction. The problem with this approach is that when many devices are trying to respond to the receiver’s transmission invitation and transmit simultaneously, a collision occurs. When the network is highly loaded, resolving such collisions is quite tedious. In this paper, we devise an enhancement to the receiver-initiated approach that aims at preventing this inherent collision scenario. Our modification relies on multiple devices sending a short predefined signal, informing their intended receiver of their intention to transmit simultaneously. The data transaction is done via a four-way handshake in which, after all backlogged devices have informed their designated receiver of their desire to transmit simultaneously, the receiver identifies them and polls them one by one, avoiding the collision. We compare the performance of Receiver-Initiated-MAC protocol (RI-MAC), which is one of the prevalent receiver-initiated protocols, with and without the suggested enhancement, and show superior air-time utilization under high traffic loads, especially in the presence of hidden terminals.

## 1. Introduction

The increasingly ubiquitous Internet of Things (IoT) penetrates more and more into the domains of our daily lives. It spans a large variety of applications, including healthcare, transportation, home automation, wearable technology, and appliances with remote monitoring capabilities (e.g., [1,2,3]). Sensors are vital enablers of the IoT, allowing a plethora of automated devices to monitor and relay collected data to a sink for further analysis and actions that need to be taken according to the collected data. Certain events can trigger additional data acquisition, either requested by the sink or automatically generated by the device, which also needs to be relayed to the sink. Accordingly, a typical IoT device incorporates two basic operations—sensing and wireless connectivity. Exploiting cheap devices enables, on the one hand, the incorporation the technology in more and more applications, yet on the other hand, it requires the development of innovative and efficient protocols and algorithms in the aspects of both sensing and communication.

One of the most prevalent means for device energy conservation is duty cycling, in which devices periodically alternate between being active and sleeping. When active, a device is able to transmit or receive data, whereas when sleeping, the device completely turns off its radio to save energy (e.g., [4]). Accordingly, one of the major challenges when operating under duty cycling is the coordination between the devices that are sharing the wireless channel and that are trying to relay the collected data to the sink. In particular, since sensors are meant to be asleep most of the time, a great challenge is to schedule a rendezvous between the transmitter and the receiver. Numerous works have suggested Medium Access Control (MAC) protocols for IoT and Wireless Sensor Networks (WSNs) under various traffic patterns and performance demands (e.g., [5,6,7,8]). These protocols can be categorized under various classification types, such as schedule access vs. random access, synchronous vs. asynchronous protocols, etc. (a short discussion is given in Section 2).

One such seminal approach was suggested by the Receiver-Initiated-MAC protocol (RI-MAC) protocol [9], in which instead of the transmitter initiating the data transaction, the receiver initiates it by sending a short preamble informing potential transmitters that it is awake and ready to receive data (an outline of the RI-MAC protocol is provided in Section 3.1). Obviously, under receiver-initiated protocols, such as RI-MAC, when multiple devices are waiting to transmit data to the same receiver, their wake-up preamble triggers simultaneous transmissions to it (resulting in inevitable collision). However, note that irrespective of the MAC protocol utilized (transmitter- or receiver-initiated), when the sink or a relay needs to collect data from many devices, the bottleneck is the wireless channel to the receiver, as many devices are trying to communicate with the same entity. Accordingly, when the surrounding devices are backlogged (e.g., in the occurrence of an event that needs to be reported), the reported data are prone to long delays and to repeated unsuccessful transmissions due to multiple devices attempting transmission simultaneously. The repeated collision problem is aggravated in the presence of hidden terminals. In this respect, contention and collision resolution mechanisms are needed to resolve this collision storm.

In this paper, we devise an amendment to the RI-MAC protocol that combats the aforementioned collision storm (The mechanism suggested herein can be applied to other MAC protocols. Nonetheless, for simplicity, and in order to isolate the mechanism from unnecessary noise, we will concentrate on RI-MAC). Our enhancement, termed Multi-Access Reservation RI-MAC (MAR-RiMAC), suggests a transmission reservation procedure that relies on multiple devices accessing the channel simultaneously. Specifically, in our modification, rather than sending the packet immediately after the receiver’s (sink’s) wakeup announcement (which results in collision when more than one device attempts transmission), each backlogged device sends a short Request to Transmit (RtR) beacon, which incorporates its identity and notifies the sink that it is backlogged. The sink identifies the backlogged devices waiting to transmit to it and polls them one by one, thus avoiding the collision. The RtR beacons benefit from two basic advantages: First, they are very short messages, and practically all they convey is the identity of their senders. Second, they can be transmitted simultaneously such that the receiver can identify the senders (the number of simultaneously transmitted RtRs supported by the enhancement is configurable and influences the length of the RtRs). We suggest two different mechanisms for the RtR realization. The first realization relies on Correlatable Symbol Sequences (CSSs), which are predefined binary codewords (sequences) that are detected by the receiver via correlators. Accordingly, each device is associated with a unique sequence that conveys its identity and is associated with a correlator; hence, it can be identified by the sink (see Section 3.3). The second realization also relies on a unique sequence associated with each potential transmitter, yet this time, rather than correlation, the decoding of the received signal relies on an energy detection mechanism (see Section 3.3). Since in both mechanisms, the number of simultaneously transmitting devices that can be identified by the sink is limited by the length of the pre-configured sequences, we devise a collision resolution mechanism for the case of the transmitting devices exceeding this limit. Our collision resolution mechanism partitions the colliding devices into smaller groups that can be supported by the multiple-access (simultaneous transmission) mechanism.

The contributions of this work are as follows:

- We devise MAR-RiMAC, an RI-MAC protocol amendment to address the collision storm problem. Our amendment is a reservation-based mechanism that allows the devices to send their reservations concurrently. Based on the received reservations, the sink pools the backlogged devices consecutively.
- We suggest two mechanisms to support the concurrent reservation transmission—one that is based on correlatable symbol sequences, and the other is based on energy detection.
- In the case that the number of concurrently transmitting devices exceeds the maximal supported concurrent transmissions, we utilize a collision resolution mechanism that distributively partitions the colliding groups into smaller subgroups, which can be supported by the concurrent reservation transmission mechanism.
- We implement both MAR-RiMAC and RI-MAC on the OMNeT++ simulator, and rigorously examine and compare the performance of both protocols under various traffic loads and topologies. We show that the suggested amendment improves performance in the case of high contention and especially in the presence of hidden nodes, yet preserves similar performance in light-traffic scenarios.

The rest of this paper is organized as follows. In Section 2, we discuss related works in data aggregation in general and duty-cycle MAC protocols for sensor networks in particular. Section 3.1 provides a brief overview of the RI-MAC protocol on which our enhancement relies. Section 3.2 describes the design of MAR-RiMAC, the suggested protocol enhancement. Section 3.3 presents two mechanisms with low communication overhead that MAR-RiMAC leverages, which enables the reception of multiple reservations simultaneously. Section 3.4, Section 3.5 and Section 3.6 discuss several properties and extensions for the protocol. Section 4 presents a thorough evaluation of MAR-RiMAC in comparison to RI-MAC via simulation results (OMNeT++ network simulator). Finally, Section 5 concludes the paper.

## 2. Related Work

The protocol suggested in this paper can apply to various setups and traffic patterns, yet it is mostly suited to the setup in which a sink (or a relay) collects data from multiple devices, and the traffic pattern is such that multiple devices have data to report at the same time; hence, it is susceptible to contention and collisions. Such a setup, in which a sink or multiple sinks collect information, such as sensed data or reports from a large population of sensors and devices, is one of the most essential tasks of Wireless Sensor Networks (WSNs) and the Internet of Things (IoT). Data collection is essential for a diverse range of tasks, such as tracking, monitoring, and surveillance, where different applications have different characteristics, specifications, and requirements. Different approaches were considered to tackle different aspects in data collection. For example, Compressive Sensing (CS) is considered to be an effective method for several aspects of data collection, such as reducing the report payload and, hence, the transmission time, or reducing the energy consumption of the sensing operation (e.g., [10,11,12,13]). Additional overviews and surveys on compressive sensing in Wireless Sensor Networks (WSNs) can be found in [14,15]. Additional means of compressions are summarized in [16,17]. In multi-hop topologies, in which the data need to travel for multiple hops before reaching the sink, attention was drawn toward load balancing and routing, as well as scheduling transmission opportunities between the relays (e.g., [18,19,20,21,22,23,24]). Under similar multi-hop-setup data aggregation protocols that fuse data arriving at a relay from multiple entities on their way to the sink, routing the data to the sink(s) has been considered (e.g., [25,26,27,28]), and a survey that compares various data aggregation techniques has been presented in [29]. Different approaches, such as compromising on the quality of the collected data, have also been considered; a survey that reviews approximate data collection algorithms can be found in [30].

One of the most important and challenging aspects of wireless communication in general and data collection in particular is air-time utilization. Since all devices communicating with a sink share the same channel (or channels), it is highly important to utilize it (or them) efficiently, all the more so because many of the devices are simple and cannot run sophisticated procedures and algorithms. Different approaches have been adopted to improve channel utilization and reduce air-time usage. Obviously, methods that compress the data—thus shortening the transmission time—such as some of the methods described above, also improve air-time utilization. In this paper, we focus on another important means to improve channel utilization, which coincides with the schemes mentioned above: the MAC mechanism, which controls the usage of the physical transmission medium. The MAC protocol utilized for data collection is highly important and affects the performance in many aspects (e.g., energy consumption, delay, reliability), especially when the network is dense and the data need to be collected from a large device population.

Numerous MAC protocols have been suggested for duty-cycle-based wireless sensor networks (e.g., [5,31]). Even though many of these inclusive MAC protocols, which were designed to handle diverse traffic patterns, can comply with report acquisition applications, their inept adaptation can result in poor performance (e.g., high latency, low channel utilization, stall reports, etc.) when the network is dense or when the traffic pattern triggers high loads in specific regions or neighborhoods. For example, adopting a synchronous approach in which neighboring devices synchronize their duty cycles such that they are all awake at the same time intervals can be a good match, as in any case, all devices in a neighborhood need to communicate with the same sink or relay (e.g., [32,33,34]). However, allocating a short awake interval to numerous devices will result in multiple collisions, which, in the best case, will allow only a few of them to transmit in a cycle. It is important to emphasize that the multi-access reservation scheme suggested in this paper can be easily applied to synchronous MAC protocols, such as DW-MAC [34], in which multiple devices can reserve transmission simultaneously, which will be scheduled during the sleep interval. Adopting the traditional transmitter initiation approach, in which the transmitter transmits a long preamble capturing the channel prior to the data transmission, waiting for its intended receiver to wake up (e.g., [35]) or breaking this long preamble to a sequence of short preambles, thus allowing the intended receiver to notify that it is awake [36,37], works fine when the traffic is sparse. However, in highly loaded regions or in dense networks in which many devices occasionally need to transmit at the same time, not only can the channel utilization be very low—kept busy with preambles for long time intervals when waiting for the intended receiver (e.g., the sink) to wake up—but also, after a device has captured the channel, the reporting devices waiting to transmit will need to defer their reports for several cycles before they can capture the channel.

Another asynchronous duty-cycle MAC approach is the receiver-initiated paradigm, in which rather than the transmitter initiating the data exchange, the receiver, whenever it is ready to receive data, invites the data exchange (e.g., [9,38,39,40,41,42]). Specifically, in the original Receiver-Initiated-MAC protocol (RI-MAC) [9], whenever the receiver wakes up and is ready to receive data, it transmits a predefined preamble to initiate data transmissions to itself (a more detailed description of RI-MAC is given in Section 3.1). Several enhancements and modifications to RI-MAC have been suggested in the literature. For example, RIVER-MAC [38] suggests two enhancements to RI-MAC. The first aims at reducing the idle listening interval—instead of a sender idly listening, waiting for a beacon from its intended receiver, a sender strobes Clear Channel Assessments (CCAs) until it detects activity. The second enhancement aims at preventing other receivers from taking control of the channel when an already active receiver invokes the collision resolution mechanism, and is specifically in a silent backoff interval. This is achieved by having the active receiver transmit a train of regular beacons, rather than remaining silent, during the backoff intervals of the collision resolution process. The first enhancement is unrelated to the enhancement that we suggest in this study (MAR-RiMAC), and can be integrated into it. The latter, which deals with the contention mechanism and combats the possible collisions during the backoff idle periods, is redundant for MAR-RiMAC, which practically eliminates the idle time intervals during contention. PW-MAC [43] and AP-MAC [39] aim at reducing the time that a transmitter is awake and waiting for its receiver to wake up by learning its receiver’s expected wake-up time. Thus, a device wishing to transmit data, instead of staying awake waiting for its designated receiver to wake up, wakes up just prior to its intended receiver’s wake-up instance. Predicting the receiver wake-up instance is again orthogonal to the MAR-RiMAC enhancement, and can also be applied to MAR-RiMAC, reducing the duty cycle of the transmitting devices (see the remark about the transmitter waking up just prior to its intended receiver on p. 24). DURI-MAC [40] is another asynchronous-receiver-initiated duty-cycled MAC protocol that, similarly to previous studies (e.g., [44]) utilizes two channels for communication: one for control and the other for data. While receiving data on the data channel, DURI-MAC suggests sending a busy signal on the control channel to avoid hidden terminals interfering with transmission. The scope of this paper is a single channel. Obviously, using part of the bandwidth to transmit a busy signal has its advantages, yet reduces the transmission rate in the data channel (narrower bandwidth). Furthermore, note that even though DURI-MAC can reduce the collisions between a data transmission after a successful handshake and a wake-up beacon by a hidden device, it cannot resolve the collision problem discussed above. In particular, the control channel enhancement suggested by DURI-MAC will not resolve collisions when multiple transmitters are waiting for the same receiver to wake up, and moves to the data channel to transmit their data after the wake-up beacon. The control channel utilization can be integrated into MAR-RiMAC, thus resolving the same kinds of collisions between data and wake-up beacons as DURI-MAC. Another asynchronous-receiver-initiated duty-cycled MAC protocol is A-MAC ([41]), which reduces the time in which a device needs to determine whether to stay awake or go to sleep after probing the channel. Since a device probes the channel numerous times a day, even a small reduction can lead to high cumulative savings in wake-up time. A-MAC further achieves more accurate decisions with respect to the staying-awake decision, reducing both false positives and false negatives. The scheme suggested by A-MAC can be applied to MAR-RiMAC. Generally, MAC protocols in which multiple nodes try to initiate a transmission simultaneously are prone to collisions and performance degradation. Although, the receiver-initiation-based approach works extremely well when, at each time instance, different sensors need to communicate with other sensors, when multiple devices need to communicate with the same receiver simultaneously (e.g., for event-driven reports to a specific sink or relay), it can perform poorly due to repeated collisions between devices trying to transmit to the same node. For example, imagine a receiver-initiated MAC protocol (e.g., RI-MAC [9]) in which a sink receives reports for numerous directly connected devices. In such cases, whenever the sink transmits the predefined signal (beacon) in order to initiate report transmissions, there is a high probability that multiple devices are waiting to transmit their reports and will start transmission simultaneously, resulting in collision. Even though receiver-initiated MAC protocols, such as RI-MAC, have devised collision resolution mechanisms to resolve such collisions (some of which are discussed above), when the number of contenders is large, resolution of such collisions with traditional mechanisms, such as the traditional backoff mechanism (e.g., RI-MAC), can be prolonged due to repeated collisions.

Some protocols address the superfluous energy consumption of a device staying awake waiting for its intended receiver to wake up (both in the transmitter- and receiver-initiated paradigms) by relying on each device (receiver) to have a predetermined wake-up schedule that is known to its intended transmitters so that they can adjust their wake-up time for transmission accordingly (e.g., [39,43,45,46]). Obviously, even though such an enhancement can reduce the duty cycle of both approaches considerably, it will not resolve the collision storm problem under the receiver-initiated paradigm discussed earlier, since all the devices waiting to transmit will wake up and respond (transmit) to the same inviting beacon simultaneously.

In this paper, in order to address the aforementioned collision storm, we suggest a highly efficient multi-access reservation enhancement, in which multiple nodes can simultaneously notify the intended receiver of their desire to transmit to it. The suggested mechanism can be easily integrated with many of the aforementioned protocols. However, for simplicity, and since the suggested enhancement is highly suited for receiver-initiated MAC protocols, we will concentrate on RI-MAC ([9]).

## 3. Multiple-Access Reservation Receiver Initiation MAC (MAR-RiMAC)

In this paper, we suggest an enhancement that allows multiple devices to inform their intended receiver (or receivers) of their intent to transmit to it (to them) simultaneously. The suggested enhancement can be applied to various protocols. However, since we want to present a full protocol and since the suggested collision avoidance enhancement is highly suited for receiver-initiated protocols, and particularly the RI-MAC protocol, we exploit RI-MAC and integrate our enhancement into it. Accordingly, we start with a brief overview of the RI-MAC protocol. The full description of RI-MAC can be found in [9].

### 3.1. RI-MAC Overview

**Figure 1:**
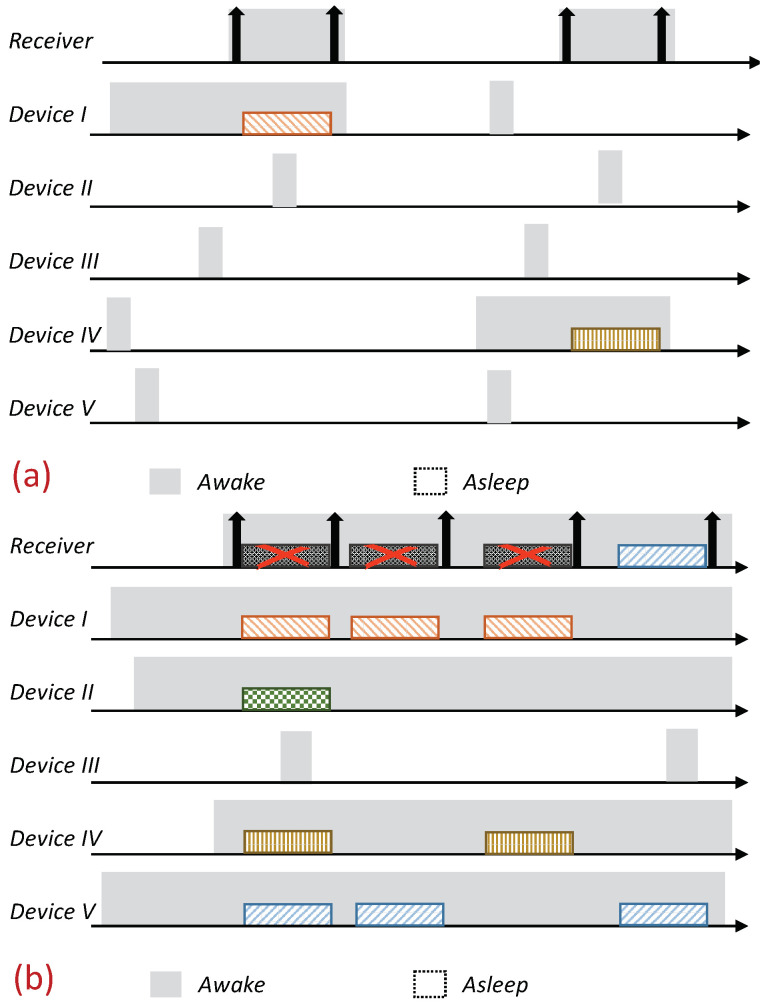
Overview of the Receiver-Initiated Medium Access Control (MAC) (RI-MAC) protocol. A backlogged device waits silently for its intended receiver beacon to start its data transmission. A non-backlogged device wakes up periodically, broadcasts its wakening notification, and, if no device transmits to it, goes back to sleep. (**a**) Under low load. (**b**) Under high load, when multiple devices attempt transmission simultaneously, a collision resolution procedure is invoked.

Receiver-Initiated MAC (RI-MAC) is an asynchronous duty-cycle MAC protocol for wireless sensor networks that aims to operate efficiently over a wide range of traffic loads. As the name suggests, in RI-MAC, the receiver initiates the data exchange. Accordingly, a node intending to send data stays active, silently waiting for its intended receiver to notify that it is awake and ready to receive data. Each node periodically wakes up based on its own schedule, and once carrier sensing the channel to ensure the medium is idle, it broadcasts a transmission invitation beacon, which is termed a “Ready to Receive” (RtR) beacon. This transmission-invitation-beacon frame contains, in addition to ordinary control fields (e.g., preamble, length), a source ID field (Src), which identifies the RtR’s transmitter. A device waiting to transmit to the awakening device (receiver), upon receiving the RtR’s handshake signal, starts its data transmission, which will be acknowledged by another beacon. The acknowledgment beacon sent by the receiver is another RtR, as, in addition to acknowledging the receipt of the data frame sent, also serves as a new RtR, inviting additional data frame transmissions to the same receiver. When no additional data frames are received, the device (receiver) goes to sleep. A basic overview of the operation of RI-MAC is depicted in Figure 1. Since when two or more devices are waiting to transmit to the same receiver, a collision will occur, accordingly, RI-MAC has devised a collision resolution mechanism. According to this mechanism, whenever a collision has occurred (the receiver has received a frame that it cannot decode), the receiver broadcasts an additional RtR in which it includes a contention window size that should be used by all contending devices such that each device should randomly select a backoff time within this window and attempt transmission only if the channel has remained idle throughout this interval. After each additional collision, the receiver generates additional RtRs in which it increases the contention window size incorporated within the RtRs (similarly to the binary exponential backoff mechanism). Note that when some of the transmitting devices are hidden from one another, the collision mechanism scheme can cause repeated collisions even when the contention window determined by the receiver is relatively large (Figure 1b).

In the following subsection, we devise an enhancement for RI-MAC that permits multiple devices to notify their intended receiver of their intention to transmit to it simultaneously, which, in turn, can pool the backlogged devices, one by one.

### 3.2. Multiple-Access Transmission Reservation Enhancement—MAR-RiMAC

As previously explained, when multiple devices are trying to transmit to the same device, as typically occurs in data-gathering applications, the performance of RI-MAC can suffer a significant performance degradation due to the multiple collisions between devices that are trying to transmit data, especially when some of the transmitting devices are hidden from one another. An illustration is depicted in Figure 1b. Our enhancement is aimed at eliminating the collisions and the aggravating consequences of these collisions, e.g., wasted air-time due to failed transmission attempts and the cost of initiating a collision resolution procedure. Accordingly, our design relies on a reservation mechanism in which even when multiple devices are trying to reserve the channel for transmission simultaneously, their intended receiver (sink) will be able to identify them and poll them consecutively. Specifically, our design relies on allocating each device a unique sequence, such that these sequences enable the receiver to identify the transmitter even during overlapping transmissions. We start by describing an overview of the protocol, assuming the existence of the mechanism that enables the receiver to decode multiple reservations transmitted simultaneously. We postpone the discussion regarding the mechanism itself to the following subsection, in which we will present two different mechanisms that can attain the required outcome.

**Figure 2:**
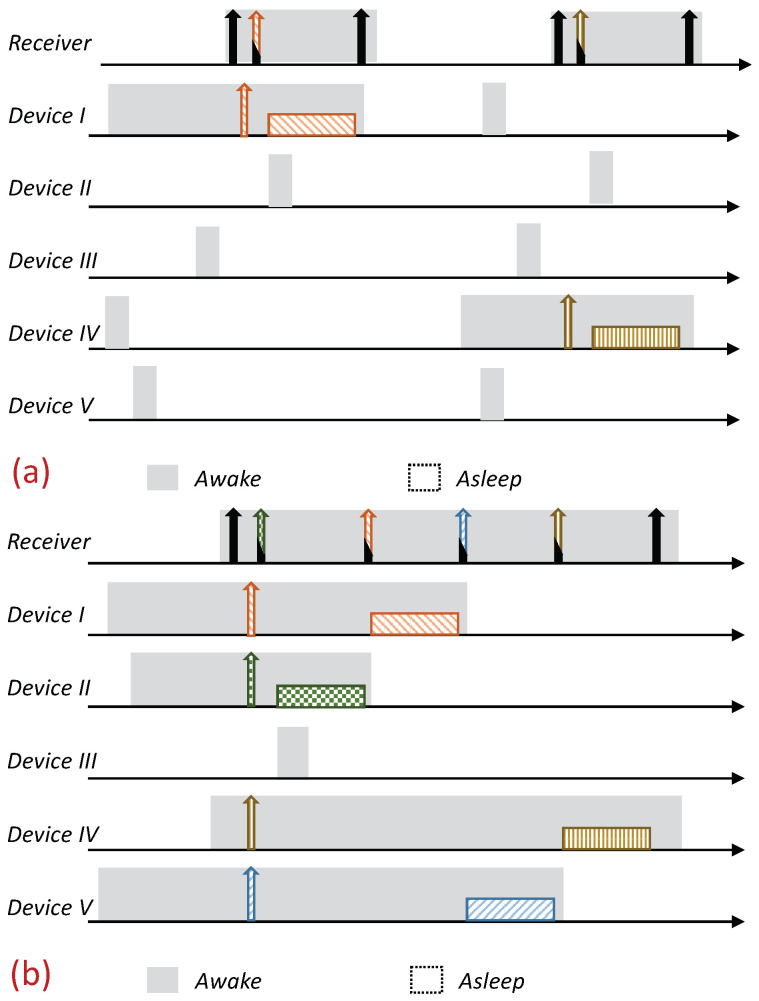
Overview of the Multiple-Access Reservation Receiver Initiation MAC (MAR-RiMAC) protocol. A backlogged device waits silently for its intended receiver beacon to send a reservation transmission. The sink sequentially polls the backlogged devices for transmission. Similarly to RI-MAC, a non-backlogged device wakes up periodically, broadcasts its wakening notification, and, if no device transmits to it, goes back to sleep. (**a**) Under low load. (**b**) Under high load, when multiple devices attempt transmission simultaneously, the sink can identify them and poll them consecutively.

The protocol is based on RI-MAC; a node with pending data (report) to send stays awake, silently waiting for its intended receiver to wake up. Each device wakes up based on its own schedule and, after verifying that the channel is idle (i.e., performing a Clear Channel Assessment (CCA check)), transmits an RtR beacon. Nonetheless, in contrast to RI-MAC, devices with pending data send a preassigned “Request to Transmit” beacon (RtT) to this awakening device (receiver). These RtTs are uniquely assigned to the devices, and can hence identify the transmitting devices distinctively. The main advantage of these RtTs is that they can be recognized by the intended receiver, even during simultaneous transmissions (the description of the RtTs is given in the following subsection). The receiving device identifies the backlogged devices that are waiting to transmit to it based on the received RtTs, and sequentially pools them for transmission. Similarly to RI-MAC, the pooling request also serves as acknowledgment of the previously received report; accordingly, the pooling request is done by sending an ordinary RtR with the pooled device ID on it (note that adding an additional address to the RtR is supported by the RI-MAC beacon frame format). An illustration of the protocol is given in Figure 2.

Next, we discuss the RtT mechanism.

### 3.3. “Request to Transmit” (RtT) Signaling

The reservation mechanism suggested above relies on the capability of the receiver of identifying the reserving devices even when it has received several reservations simultaneously. Accordingly, our goal is to introduce a robust transmission mechanism with low communication overhead, which enables the reception of multiple transmitted signals simultaneously. In this subsection, we will present two different mechanisms that can attain this goal.

#### Correlatable Symbol Sequences (CSSs)

**Figure 3:**
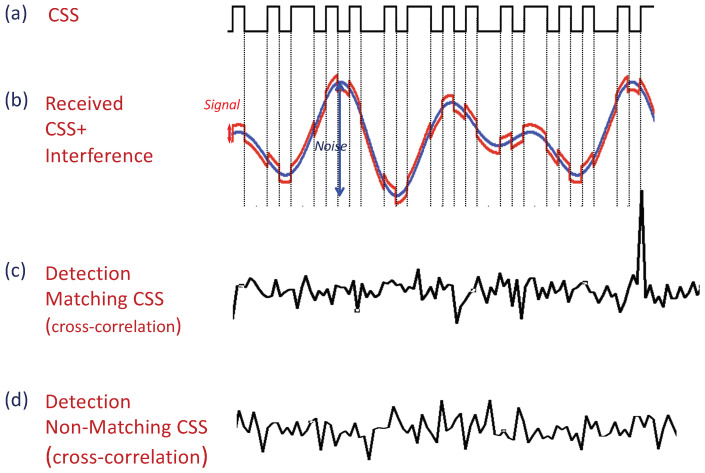
A device utilizes Correlatable Symbol Sequences (CSSs) to convey its identity. The receiver (sink) detects the specific CSS by cross-correlating it with the incoming samples. (**a**) CSS- predefined binary codeword (sequence). (**b**) CSS+Interference as received by the receiver (sink). (**c**) CSS detection process via cross-correlation. The receiver (sink) cross-correlates the incoming samples with the pre-defined CSSs. Cross-correlating with a matched CSS obtains a spike value. (**d**) Cross-correlating with a non-matched CSS does not produce spike values.

**Figure 4:**
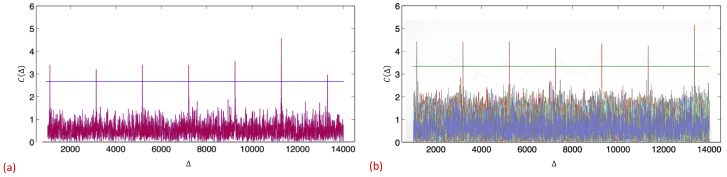
A sender conveys information to the receiver via a pre-defined CSS. For each newly acquired sample, y(k+Δ), the receiver computes the signal’s correlation with a local copy of a particular CSS, $C\left(\Delta \right)=\sum_{k=0}^{L-1}s^{*}\left(k\right)y\left(k+\Delta \right)$, where $s^{*}$ denotes the complex conjugate of the target CSS: *s*. The correlation value spikes every time a pre-defined CSS is received. (**a**) Signal correlation of seven repetitions of a 127-symbol-length Gold-code codeword under background interference, generated by random OFDM transmissions of a potential background interference (received signal to interference and noise ratio (SINR) is −6 dB). (**b**) Cross-correlation values of a specific CSS when an additional 10 different CSSs were sent. The figure depicts the cross-correlation values with each of the 11 CSSs. The correlator spikes when the designated CSS is received. It is low for all ten other CSSs. Note that the cross-correlation values stay low until y(Δ) is aligned with the beginning of *s*.

The first mechanism utilizes Correlatable Symbol Sequences (CSSs). Correlatable symbol sequences are predefined binary codewords (sequences), which are detected by the receiver via correlators. Accordingly, each codeword is associated with a correlator, which is essentially an array of *n* weights, where the size *n* depends on the desired number of codewords and the robustness of transmissions. Even though these codewords are deterministically generated, they retain the statistical properties of a sampled white noise, according to their designation as pseudo-random or pseudo-noise (PN) sequences. While these appear random to an ignorant listener that has no prior knowledge of the predefined codewords, PN can be easily detected by the legitimate receiver via cross-correlation with a local copy; cross-correlation of any such sequence with a matching copy obtains a sharp and distinct spike when the input signal to the correlator matches the PN sequence itself. Correlation-based detection with a pseudo-random sequence makes the detection more reliable and robust to noise and interference, i.e., CSSs can be detected even at a low signal to interference and noise ratio (SINR). Figure 3 illustrates the codeword detection process. A detailed discussion of signal correlation can be found in the literature (e.g., [47,48,49,50]). For our CSS mechanism, we utilize sequences with low cross-correlation, which means that any two sequences within the set have bounded small cross-correlations. Accordingly, trying to cross-correlate any sequence with any other but its own corresponding sequence (legitimate or not) will result in a correlation close to zero (e.g., Gold and Kasami codes, which are commonly used; e.g., [48,51]). CSSs are widely used in communication systems (e.g., customary IEEE802.11 preamble used for packet detection, symbol synchronization, and radio parameter tuning). In order to illustrate the CSS concept and, specifically, the correlation and cross-correlation advantages, we present previous experiments that we have conducted on the Wireless Open-Access Research Platform (WARP) (a comprehensive set of the experimental results that examine different aspects of CSSs and can be found in [52,53]). Here, we present two examples that are borrowed from there. Figure 4a depicts a representative outcome of a realization in which the sender utilized 127-symbol-length Gold codes for CSSs. It transmitted seven repetitions of a 127-symbol CSS. We evaluated the detection of a CSS codeword under background interference generated by random orthogonal frequency-division multiplexing (OFDM) transmissions of a potential interfering node. For each newly acquired sample (at 40 MHz frequency of the WARP platform analog-to-digital converter (ADC)), the receiver computes the signal’s correlation with its local copy of the transmitted CSS. The x-axis in both figures represents the index of the collected samples. The y-axis represents the sliding window value attained by the correlator for each received symbol. The horizontal line represents a possible threshold, which is configurable and balances between false positive (too low) and false negative (too high) detections, thus determining the robustness of the CSS. Figure 4a demonstrates the robustness of the correlation process. As can be seen in the figure, the reception of each CSS results in a correlated spike that is clearly identifiable, indicating that CSSs can be successfully received even under low SINRs (−6 dB). Figure 4b depicts an overlap of the outcome at the receiver of eleven different correlators (correlation with eleven different CSSs, including the genuine one). Again, it can be seen that the mechanism is highly robust and the receiver can easily detect the transmitted CSSs, while the other correlators did not produce any false negatives, despite the very low SINR (i.e., low cross-correlation between the set of CSSs). Note that the noise only looks visually denser due to overlapping plots.

The utilization of a CSS detection process via cross-correlation as our signaling mechanism enjoys three fundamental advantages over the ordinary contention mechanism: (i) It enables a reliable signaling mechanism between the reporting devices and the report accumulator even at low SINR. (ii) Short-duration signals (transmission times) convey the signaling device’s ID. Note that the duration of a signal CSS transmission, overlooking the simultaneous transmission gain, is much shorter than a typical control message duration, especially since a typical control message is proceeded by a CSS. Furthermore, the detection is almost instantaneous, as no decoding is needed. (iii) The benefit from CSSs being transmitted simultaneously (multiple CSSs at a time) is two-fold; first, simultaneous—rather than sequential—transmission saves valuable time. Second, the fact that collisions are avoided not only saves the wasted air-time due to unsuccessful transmissions, but also enables the signaling devices to transmit right after the RtR beacon without any contention delay.

The sequence length determines the transmission duration of each CSS as well as the number of supported sequences, which, in turn, determines the probability of misdetection and the number of correlators required at each report aggregator. It is important to note that CSS correlators require a limited amount of resources (they require sign-flip adjustments and several summations on the received samples). Moreover, since correlators are already realized in common wireless cards, CSS enhancement only requires replication of components (all the more so since, basically, only the sink needs to attain many correlators). Obviously, there is a tradeoff between the number of supported sequences and the probability of misdetection, i.e., the length of the sequences in conjunction with the number of supported CSSs determines the magnitude of the cross-correlation between any CSS pair, which, in turn, influences the probability of false detection. PN sequences have been widely explored in the literature; each is characterized by the sequence length (i.e., number of symbols), the number of sequences that can be generated (in our context, how many different signals or different IDs can be supported), and the cross-correlation between any sequence pair, which is an indication of accuracy (misdetection). For example, our suggested scheme can utilize a Gold code with a 127-symbol sequence length, which can support 127 CSSs with minimal misdetections (practically none). Another alternative is the large set of Kasami sequences with 255 symbols per sequence, which can support 4011 CSSs with similar performance (e.g., [48]).

**Figure 5:**
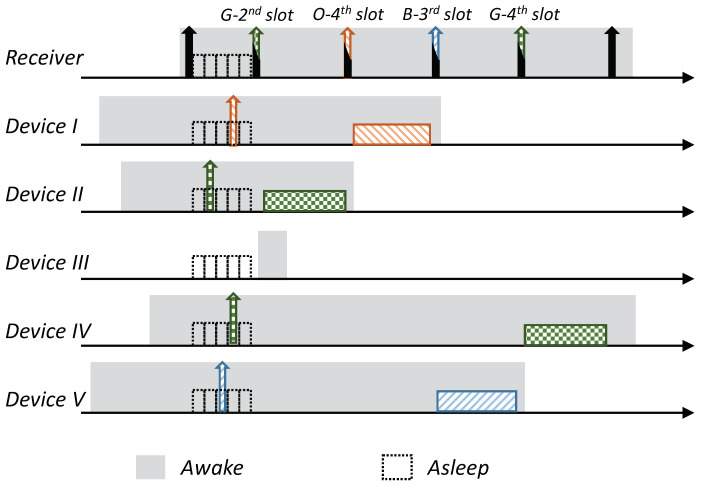
Reuse of CSSs by multiple devices in the vacancy via a small contention interval. Device II and Device IV utilize the same CSS, yet choose different minislots; hence, they can be distinguished by the sink. Device I and Device IV choose the same minislot, yet utilize different CSSs; hence, they can also be distinguished by the sink.

If the number of devices is very high, instead of extending the length of the CSS sequences to support a unique sequence for each device, which increases the transmission overhead even under light loads, we can reuse CSSs. Specifically, since the devices usually send reports only occasionally, not all the devices in the neighborhood are expected to transmit a report at the same time, and only a subset of devices is expected to transmit simultaneously; the number of required CSSs can be reduced based on this expected number, i.e., devices will not receive unique CSSs and they will have to choose from a common CSS pool. Obviously, reducing the number of CSSs also reduces the sequence length, i.e., transmission duration. However, since multiple devices utilize the same CSS pool and multiple devices can use the same CSS, we need to find a mechanism to associate a received CSS with the device that sent it. If the number of CSSs is much higher than the expected number of devices that may transmit simultaneously, we can let each device choose which CSS to use at each transmission opportunity randomly by relying on the low probability that different devices will choose the same CSS (e.g., with 127 CSSs and five devices transmitting simultaneously, the probability that each device will choose a different CSS is over 0.92). In this case, the sink will pool the devices based on the CSSs used (i.e., the index or some other CSS identification will be included in the polling message). Note that if two devices choose the same CSS, there will be a collision while transmitting their reports, yet the rest of the devices that chose a unique CSS will have no problem in sending their reports; the colliding devices will resolve the collision according to the collision resolution mechanism described below after randomly choosing a different CSS. Another option is to define a small contention interval starting right after the RtR message, such that each device randomly chooses the CSS and the timing (minislot) with which it transmits its reservation request. The sink will pool the device based on the CSS and the timing; now, collision will occur only if two devices choose the same CSS and the same time slot. An illustration of a contention interval with five minislots is given in Figure 5.

Aggregated Signal Collection (ASC): The second mechanism that can be utilized relies on group testing [54]. It also relies on each device transmitting a unique binary predefined sequence after receiving the RtR signal. Specifically, each device is assigned a unique sequence of ones and zeroes of length *T* (the value of *T* will be determined later). Exactly as before, whenever a device wants to transmit a report, it waits for the inviting RtR in order to signal the sink that it is backlogged (has a report to send). Each RtR is followed by *T* conceptual minislots; each minislot duration is sufficient for the sink (report aggregators) to receive a signal from the furthest device, with sufficient time-guard boundaries such that there are no overflows to the sequential slot (starting at the current slot and leaking into the next slot). Immediately after receiving the RtR, a backlogged device, which is awake and waiting to transmit, transmits its transmission reservation indication (RtT) by following the pattern indicated by its exclusive sequence, such that the device transmits at each minislot that corresponds to it, which is 1 in its sequence, and it remains idle if it is 0. We denote minislots in which at least one of the active (signaling) devices is transmitting (the respective minislot index in its sequence is “1”) as “Active”, and minislots in which none of the signaling (waiting to report) devices are transmitting (the respective minislot index in all signaling devices’ sequence is “0”) as “Idle”. After receiving the *T* minislots, the sink can identify the signaling devices merely based on the “Active” and “Idle” minislots. Note that consequently, it is sufficient for the sink to rely on a simple energy detection mechanism, which is sufficient to identify which minislots are “Active” and which are “Idle”. After identifying the signaling devices, the sink pools their reports sequentially one at a time.

The length of *T* and the construction of the code (the unique pattern assigned to each device) are determined based on the maximal number of devices that the sink can identify even when they signal simultaneously. Specifically, the mechanism is designed to support the maximal number of simultaneous transmissions. We denote this number by *K*. In the event that the actual number of signaling devices exceeds *K*, i.e., more than *K* devices are trying to signal simultaneously, the sink will not be able to identify the signaling devices, and a collision resolution mechanism should be invoked (the collision resolution procedure will be discussed in the sequel). This number of maximal supported simultaneous transmissions (*K*) should be configured based on the topology, the application that the devices are performing, the performance constraints, and the device capabilities. The higher the *K*, the greater the signaling overhead per se (i.e., the higher *T*), yet the smaller the collision probability (i.e., the greater the probability that more than *K* devices are trying to signal simultaneously). Accordingly, *K* should be chosen such that it balances between the signaling overhead and the collision probability, which requires invoking the collision resolution procedure, which also incurs waste of resources (overhead).

The devices’ sequences should be designed such that the sink will be able to identify the active devices provided that the number of active devices does not exceed *K*. It has been shown that an efficient choice of parameters should be such that the ratio between the “Active” minislots (i.e., at least one of the active devices has 1 in its sequence and is, hence, transmitting) and the “Idle” minislots (i.e., all active devices have 0 in the respective minislot index in their sequence; hence, none are transmitting in this minislot) should be $\frac{1}{2}$ irrespective of the actual active devices, i.e., for any set of *K* active devices (e.g., [55]). The required number of minislots (i.e., sequence length) that is sufficient to identify the set of active devices is known to be in the order of T=O(KlogN), in which *N* denotes the total number of devices (e.g., [55,56]). Obviously, this number can be slightly increased in order to reduce the error probability. It was shown that the sequences themselves can be constructed randomly such that each entry (bit) in each sequence is determined according to the Bernoulli distribution with parameter $p=1-2^{\frac{-1}{K}}$. The maximum likelihood (ML) decoder, which picks the best-matched set of devices for potentially generating the binary outcome, can be used to identify the signaling set. In addition, several more efficient decoding algorithms—at the expense of slightly longer sequences—are proposed in the literature for identifying the signaling devices (e.g., [55,56]).

### 3.4. Collision Resolution

**Figure 6:**
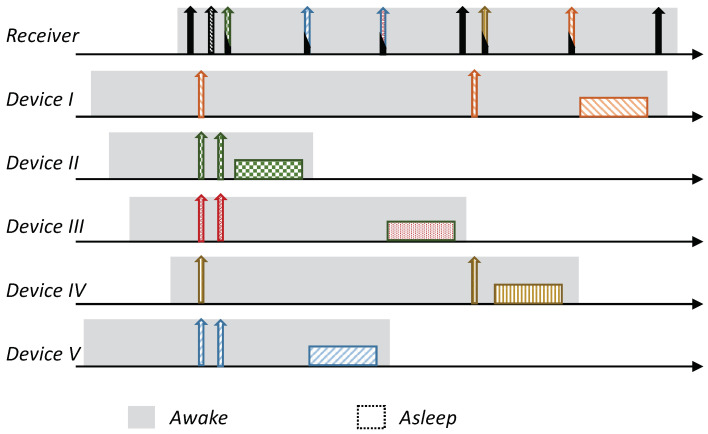
MAR-RiMAC collision resolution procedure illustration for K=4. After the sink’s “Ready to Receive” (RtR) beacon, five devices (more than K=4) attempt transmission. The sink sends a “Ready to Receive Retransmissions” (“RtRR”) to resolve the conflict, which is replied to by three devices (less than K=4). The other two devices transmitted after the sink finished polling the three devices and sent a new RtR.

As previously explained, the sequences are designed to cope with a maximal number of simultaneous transmissions (*K*). If more than *K* devices are trying to signal simultaneously, the sink will not be able to exclusively identify the signaling set (i.e., collision). Accordingly, in case the sink cannot identify the report-sending devices, it initiates a collision resolution procedure. The collision resolution protocol is based on the binary-tree Collision Resolution Protocol (CRP) (e.g., [57], Chapter 5). Specifically, after the sink realizes that it cannot identify the devices that have sent the RtT, it generates a designated “Ready to Receive Retransmissions” beacon (denoted by “RtRR”), indicating that all the contending (signaling) devices should participate in the collision resolution procedure. Obviously, all these devices are awake and waiting to be polled for their reports. After receiving an “RtRR”, each of the colliding (reporting) devices draws a random decision on whether to signal again or stay idle. Explicitly, they signal again with a probability of 0.5 and stay idle otherwise. The devices that choose to wait (draw to stay idle) wait for all the devices that have chosen to transmit their sequence (draw to signal) to be polled for their reports, and, only after, try again to transmit their signals. Specifically, immediately after the sink finishes polling the reports of all devices that signal after the “RtRR”, it transmits another “RRtR” for which all the devices that were involved in the previous collision, yet stayed idle after the previous “RtRR”, transmit their sequences. The process repeats recursively such that in the case that, after “RtRR”, more than *K* devices have decided to signal (more than *K* draw to transmit and, hence, the sink still cannot identify the signaling devices) the sink retransmits another “RtRR” for which only the devices that participated in this last failed attempt (collision) redraw and, with a probability of 0.5, retransmit or stay idle otherwise. Since the recursive process follows a tree structure in which each branch terminates by no more than *K* devices having transmitted their sequences, all the devices that participated in the first collision can track down the progress and determine their turn to retransmit their sequences. Note that the recursive process resembles the binary-tree CRPs; however, in contrast with these protocols, in which each branch of the tree terminates with no transmitting devices or a single transmitting device, now, each branch terminates with no more than *K* devices transmitting their reports. Figure 6 illustrates an example of the collision resolution procedure for K=4. Note that after the sink’s awakening RtR beacon, five devices attempt transmission. Since the sink cannot identify them, it sends an “RtRR”, for which three devices have transmitted “RtT”. After pooling the three devices, the sink sends another RtR beacon for which the two other devices can transmit their “RtT” signals and be pooled consequentially.

Remark I: Even though the procedure applies to any *K*, it is more efficient when the number of devices expecting to contend (with a report to send at a specific time epoch) is much lower than the overall number of devices (which is expected to be very high), i.e., it is most efficient when K<<N (in which *N* denotes the total number of devices). Note that this proportion is very realistic, as we expect the network to be dense, with a lot of reporting devices, yet since each device is expected to wake up and send a report only sporadically, we expect the number of waiting-to-transmit devices at a particular time to be relatively small. Obviously, the set of waiting-to-transmit devices changes at each transmission opportunity based on each device’s objective, expected report frequency, duty cycle, etc. Note that when *N* is high and K∼N, other procedures, such as Binary Exponential Beacons (BEBs), work poorly. In the aforementioned discussion and, in particular, in the random sequence discussion, when determining the Bernoulli distribution parameter, we have assumed that $K\leq N^{\frac{1}{3}}$. For greater *K*, a different Bernoulli distribution parameter should be chosen [55].

Remark II: Even though, at first glance, it might seem that the ASC procedure is identical to the CSS mechanism, it is important to note that the two procedures are different, not least because the latter relies on correlators, while for the former, it is sufficient to rely on energy detection, which identifies which minislots are “Busy” and which are “Idle”. Note that the cross-correlation between different sequences in the ASC scheme is not negligible. Trying to correlate a sequence of a non-transmitting device with the outcome will not produce a near-zero outcome. On the other hand, it is sufficient that only one bit of a non-transmitting device is 1, while the corresponding minislot at the outcome is “Idle”, i.e., none transmitted at this minislot, indicating that the device was not transmitting.

### 3.5. Robustness to Noise

As previously explained, the CSS approach is highly robust to noise, i.e., CSSs can be successfully detected even under very low SINRs (−6 dB). The ASC approach is also resilient to noise, as it only relies on a simple energy detection mechanism that only requires distinction between “Busy” and “Idle” minislots, i.e., to determine whether the received signal is above a predefined threshold. Note that similar detection is utilized by many protocols that rely on a carrier sense mechanism that needs to determine whether the received signal is above the noise floor (“Busy”) or not (“Idle”). It is still possible to have minislot misdetections, which can be split into two types: false positive, in which the sink labels the minislot as “Busy” even though no device was transmitting in the minislot, or false negative, in which the sink labels a minislot as “Idle” even though at least one device has transmitted in this minislot. The former error is more common due to noise spikes or unexpected interference. Resiliency to such errors can be attained by extending the sequences and, in particular, adding sufficient redundant bits (which result in additional minislots) to ensure that the actual signaling set is discovered despite the few erroneous minislots. The longer the sequences, the less susceptible to noise, thus trading overhead for robustness. An analysis of the number of redundant bits that are required to render the ASC insusceptible to noise can be found in [58].

### 3.6. Multi-Hop Topologies

Throughout this paper, we concentrate on a single-hop topology, in which a sink collects data from many devices. The suggested mechanism can be applied to multiple-hop topologies, in which data from different devices need to travel for multiple hops before reaching the destination. Recall that RI-MAC was designed to cope with multiple-hop topologies; accordingly, the procedure suggested for a single sink collecting data directly from many devices can be applied to each relay along the way, which collects data that need to be relayed from devices or from other relays. For this procedure to work, each device or relay needs to be assigned a sequence that identifies it. Preferably, the sequences should be distributed such that there are no conflicts in a neighborhood, i.e., each device and relay in the vicinity of a relay (so that the relay can receive its signal) is assigned a unique sequence. Reuse of sequences outside a neighborhood does not pose a problem. If there are no sufficient sequences to share uniquely within a neighborhood, and different devices share the same sequence within a neighborhood, there can be conflicts that need to be resolved. For example, consider two devices (or relays) sharing the same sequence that are within communication range of a relay, yet one needs to communicate with the relay while the other needs to communicate with a different relay. In the unlikely event that the relay receives an identity sequence from the device (or relay) not associated with it (which is unlikely, as it requires two relays to send inviting RtRs at the same time), the receiving relay needs to take into account that the polling message inviting the device to transmit will not be reciprocated.

As before, the multi-hop MAR-RiMAC enhancement is more appropriate in a data collection application in which a sink collects data from numerous devices, some of which are several hops away. A topology in which devices communicate with different devices is less suited for the MAR-RiMAC enhancement, as the likelihood that multiple devices will try to communicate with the same device is low; hence, the probability for collision between devices trying to communicate with the same device is low. In any case, it should be emphasized that MAR-RiMAC is transparent to the routing protocol and can support any routing protocol, or at least any routing protocol that relies on a receiver-initiated protocol.

Downstream traffic can be incorporated with the upstream traffic by utilizing the same procedure as the four-way handshake suggested by MAR-RiMAC. However, if the overlay routing topology is a tree topology, in which each device or relay receives data from only one relay, downstream traffic can be handled only via a two-way handshake, similarly to RI-MAC.

## 4. Performance Evaluation

In this section, we evaluate the performance of our protocol MAR-RiMAC using simulations. We compare MAR-RiMAC with the original RI-MAC ([9] protocol, which exploits a traditional backoff as a collision resolution mechanism. Our experiments use the same MAC layer parameters as [9].

### 4.1. Simulation Setup

We utilized the OMNeT++ network simulation environment with the INET Framework enhancement (version 5.6) to evaluate our protocol. As the connectivity model, we deployed the unit disk graph model, which assumes that each sensor node is equipped with a single omnidirectional antenna and all sensors transmit at the same power. The area within which a signal from one sensor can be received and decoded by another sensor is modeled as a circle, with a radius denoted by the transmission range. We evaluated the suggested protocol in various network densities by generating diverse scenarios comprising a single sink and a varied number of devices. The size of the deployment area was fixed, and the devices were distributed uniformly over the deployment area. We focused on topologies in which all devices were within transmission range with the sink, i.e., could transmit directly to the sink. We examined two basic topologies under this setup: In the first set of simulations, all devices were within transmission range of one another (single-hop topology with no hidden terminals). In the second set, the transmission range was set to allow hidden terminals, i.e., some of the devices were out of transmission (and sensing) range of other devices. In the latter setup, we ensured that there was at least one pair of devices hidden from one another. Since the paper concerns a data collection MAC protocol, our evaluation focuses on traffic generated by the devices destined to the sink (upstream traffic). Traffic was generated at each device according to the Poisson process with varied intensity (arrival rate), where we examined various traffic loads ranging from very high, to moderate, to very light traffic. The sleep interval was set to one second, and the initial wake-up time of each device was randomized in our evaluation. Each simulation lasted 1000 s, and was repeated at least 100 times under different seeds. The results presented herein are an average over the 100 runs. For the MAR-RiMAC, we utilized the ASC scheme that limits the number of devices that can transmit reservations simultaneously. We kept this number at 4 (K=4), which is very conservative. As discussed in Section 3.2, the number of minislots (*T*) required for the reservation process depends on the supported number of reservations taht can be transmitted simultaneously and still be decoded (*K*) and the total number of devices (denoted by *N*). The duration of each such minislot should encompass the maximum propagation delay between any device and the sink and the time required by the sink to identify that there is a transmission in progress, which can be very short, as it only corresponds to the duration required by the sink to sample the channel and identify that there is a transmission in progress (sufficient for the energy detection mechanism). Both the propagation delay in a few-hundred-meter topology as well as the energy detection mechanism are in the order of a few micro-seconds. In the simulations, we set the duration of *T* across all our simulations to be constant (the same as a preamble), which, under K=4, can support over 70 users. Note that the performance under the CSS scheme is expected to be superior to that of the ASC that is depicted below for two reasons. First, the duration of a CSS is much briefer than *T*. Second, while the number of simultaneously transmitted reservations supported by the ASC is limited by *K*, with CSSs, the number of simultaneously transmitted reservations is limited by the number of CSSs, i.e., if each device in a neighborhood is assigned a unique CSS, no reservation collisions will occur regardless of the number of simultaneously transmitting devices. For comparison, we also implemented the RI-MAC protocol ([9]). In the following, we present the results attained by both protocols. Accordingly, the main parameters are taken from the RI-MAC paper ([9]). The key parameters we used are summarized in Table 1.

In the sequel we present the results attained by both protocols.

### 4.2. Clique Topology

The first part of the simulations considers a clique topology in which all devices can sense one another (no hidden terminals). We present results for different numbers of devices (2, 5, and 20 devices) and for various traffic loads (mean inter-arrival times of 0.9, 0.95, 1, 1.35, 1.5, 2, 5, and 10 s per packet). Since the devices’ cycle interval is 1 s, lower inter-arrival times imply that the devices mostly wake up backlogged. On the other hand, an average inter0arrival time of 10 s/packet indicates a very light load in which, most of the time, there is at most one backlogged device (low collision probability), especially when the number of devices is low. In order to improve the latency, both protocols support the transmission of several packets per cycle (per wake-up), i.e., after transmitting a packet, the device checks whether it has more packets to send, and if it does, it remains awake and contends for additional transmissions. The device goes to sleep only when it is not backlogged (its queue is empty). We emphasize that this multi-packet transmission per cycle enhancement was implemented for both MAR-RiMAC and RI-MAC.

**Figure 7:**
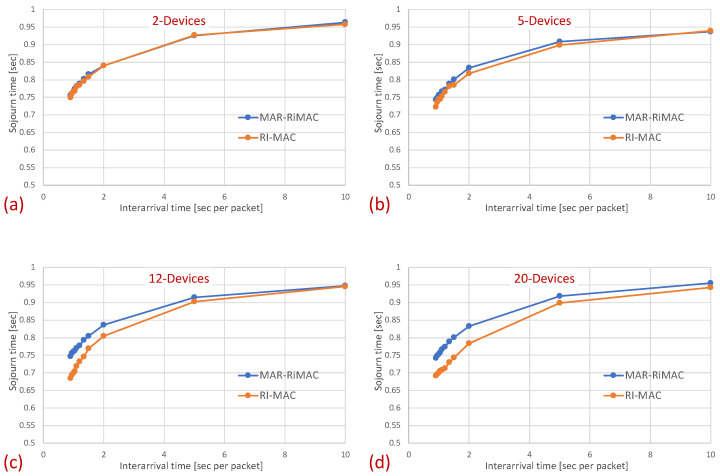
Clique topology: Mean sojourn time for different numbers of devices and maximal number of simultaneous transmissions supported by MAR-RiMAC; K=4. (**a**) Two devices. (**b**) Five devices. (**c**) Twelve devices. (**d**) Twenty devices.

Figure 7 depicts the average latency vs. the inter-arrival times for different numbers of devices.

The results clearly depict that, as far as the latency is concerned, the performance of both protocols is quite similar, with a slight advantage for the RI-MAC protocol. On the one hand, MAR-RiMAC imposes a slightly higher overhead due to the four-way handshake, yet this overhead is negligible with respect to the cycle time or even the packet transmission time. On the other hand, the contention-handling mechanism of MAR-RiMAC is less time consuming than RI-MAC’s, yet since all devices can hear one another, RI-MAC’s collision resolution mechanism is very efficient.

Note that the longer it takes the sink to resolve collisions, the longer it stays awake, which, on the one hand, increases the latency incurred by the collision resolution mechanism, yet can reduce the average latency by two means. First, the longer the sink stays awake, the more backlogged devices are waking up while the sink is awake, and they can transmit their packets before it goes to sleep, rather than waiting for the next time the sink wakes up. Second, a packet that arrives when the device is awake (e.g., contends for transmission or resolves collision) will be transmitted before the device goes to sleep, while a packet that arrives while the device is asleep will wait for the next cycle. We will re-examine this observation in the following.

Note two prominent phenomena: First, interestingly, the average sojourn time for both protocols seems quite high, especially for the low traffic loads, e.g., the average sojourn time for N=2 devices with an inter-arrival time average of 0.9 s/packet is 0.75 s for both the MAR-RiMAC and the RI-MAC protocols. However, note that packet arrival times are independent of device wake-up times. Accordingly, the time elapsing between the arrival of a packet and the next wake-up time of its respective device is, on average, half a cycle (0.5 s). Accordingly, a packet that arrives while its device is asleep will need to wait, on average, half a cycle before its device is awake. After the device is awake, it needs to wait for the sink to be awake before transmitting a packet. Since the wake-up times of the devices are chosen arbitrarily, and since the results presented herein are averaged over all the devices and over multiple runs, the elapsed time between wake-ups of a device and the sink is, on average, an additional half cycle. More interestingly, the results show that for both protocols, the average sojourn time is lower for higher traffic loads than for lower traffic loads, e.g., the average sojourn time for N=20 devices with an average inter-arrival time of 10 s/packet is 0.44 s for both the MAR-RiMAC and the RI-MAC protocols, while for an arrival rate of 1.1 packets/s (0.9 s average inter-arrival time), it is only 0.74 and 0.69 s for the MAR-RiMAC and the RI-MAC protocols, respectively. This result seems counterintuitive, as one could expect that high traffic loads result in higher collision probability with increasing numbers of retransmission attempts and, hence, higher delays. However, the fact that a device is staying awake longer allows it to receive an additional one or more packets for transmission while it is still awake, and to transmit them in the current cycle rather than delay its transmission until the next time that it is awake.

**Figure 8:**
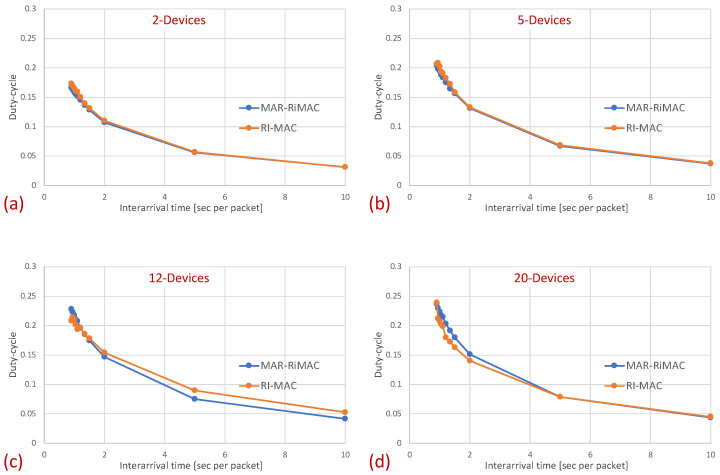
Clique topology: Mean duty cycle for different numbers of devices and K=4. (**a**) Two devices. (**b**) Five devices. (**c**) Twelve devices. (**d**) Twenty devices.

Next, we examine the average duty cycle, which presents the percentage of time that a device is awake, and hence provides an indication of the device’s power efficiency. Recall that the sleep cycle was set to 1 s; hence, a duty cycle of 5% indicates that, on average, the transceiver is active only for 50 ms/cycle. Figure 8 depicts the duty cycle results for the same set of topologies and parameters (e.g., mean arrival times) as those presented for the mean sojourn times.

When examining the duty cycle, recall that, in addition to the periodic wake-up times in which devices need to transmit their beacons (RtR beacons) to announce that they are awake and to initiate potential transmissions to themselves, they need to stay awake in order to transmit backlogged traffic to the sink. Accordingly, the larger the load, the higher the duty cycle for both protocols. Specifically, the inter-arrival time affects the duty cycle in two ways: First, the lower the mean inter-arrival time, the more packets the device needs to send and the longer the time it needs to transmit these packets. For instance, even when there is no or very little contention, each time a device needs to send a packet, it needs to stay awake waiting for the sink to wake up before sending the packet. Second, when the mean inter-arrival time is low, more devices are backlogged and, in addition, to the time waiting for the sink to wake up, they need to stay awake longer waiting for their opportunity to transmit (contention resolution time). However, as before, the longer the sink is awake, the more probable it is that a device will find it awake and will not need to wait for its next awake epoch. In addition, the longer a device stays awake transmitting packets back to back rather than distributing their transmission over multiple cycles, the shorter its awake time on average (e.g., a device transmitting 10 packets in one cycle while transmitting only its RtR beacon in the following nine cycles is less awake than a device transmitting the 10 packets at one per cycle).

In Figure 8, it is evident that for a small packet size (28 bytes), the duty cycles of both protocols are quite similar. For example, in the 20-device topology, when the average inter-arrival time is 0.9 s/packet, the duty cycle is quite high: 24% for both the MAR-RiMAC and RI-MAC. When the load is low, even for the 20-device setup, the duty cycle is quite low (for an average inter-arrival times 10 s/packet, the duty cycles are 0.04% for both protocols), as devices are typically not backlogged, and can hence go to sleep immediately after waking up and transmitting their RtRs. Whenever they are finally backlogged, it is very likely that no or only a few other devices are backlogged concurrently; hence, the contention resolution mechanism (for both protocols) is expected to terminate quite fast. In order to confirm our assertion that both the sojourn time and the duty cycle of RI-MAC benefit from the sink’s long awake period incurred by the lengthy contention and collision resolution mechanisms, we compare the sink’s duty cycle.

**Figure 9:**
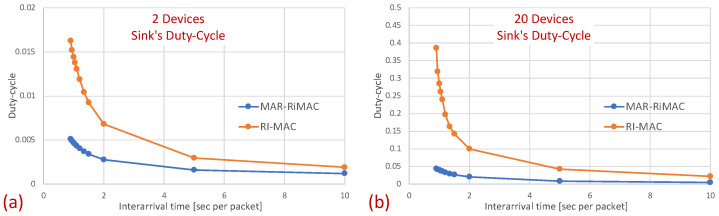
Clique topology: The sink’s mean duty cycle for different numbers of devices and K=4. (**a**) Two devices. (**b**) Twenty devices.

Figure 9 confirms that the sink’s duty cycle for RI-MAC is indeed much higher than that for MAR-RiMAC, even when the number of contenders is very low (only two devices), and the contending devices can sense one another (no hidden terminals), which implies low contention. Next, we examine the performance in the presence of hidden terminals.

### 4.3. Hidden-Terminal Topology

**Figure 10:**
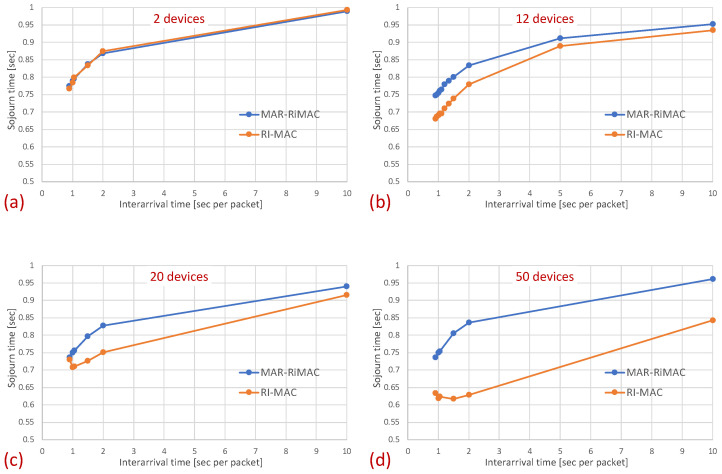
Hidden terminals: Mean sojourn time for different numbers of devices and K=4. (**a**) Two devices. (**b**) Twelve devices. (**c**) Twenty devices. (**d**) Fifty devices.

**Figure 11:**
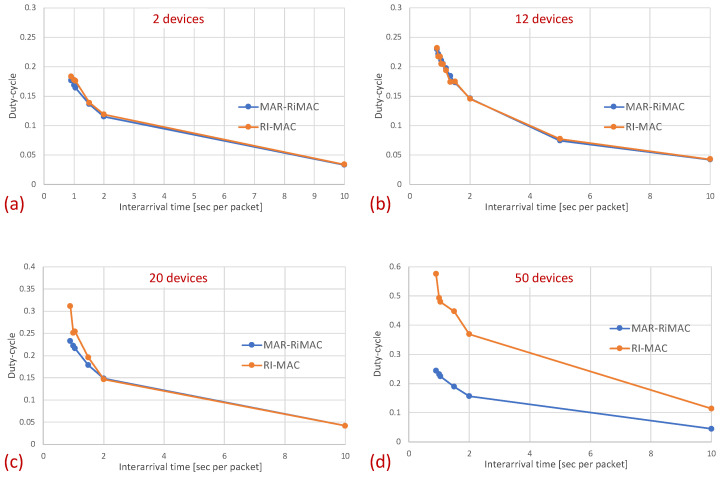
Hidden terminals: Mean duty-cycle for different numbers of devices and K=4. (**a**) Two devices. (**b**) Twelve devices. (**c**) Twenty devices. (**d**) Fifty devices.

In this part of the evaluation, we examine the protocols’ performance under hidden-terminal topologies. In order to ensure that some of the devices are hidden from one another, we have limited the interference range of each device such that it will not cover the whole area. We positioned the sink at the center and set the transmission range to be slightly larger than the distance between the center and each of the four corners of the simulation zone, such that all devices, wherever positioned, can transmit directly to the sink. We further positioned four devices around the four corners of the simulation zone such that they were in transmission range with the sink but hidden from one another; the rest of the devices were randomly scattered over the simulation terrain. As before, we examined the performance under different numbers of devices and traffic loads. The results for the sojourn time and for the duty cycle for different numbers of devices and traffic loads are depicted in Figure 10 and Figure 11, respectively.

**Figure 12:**
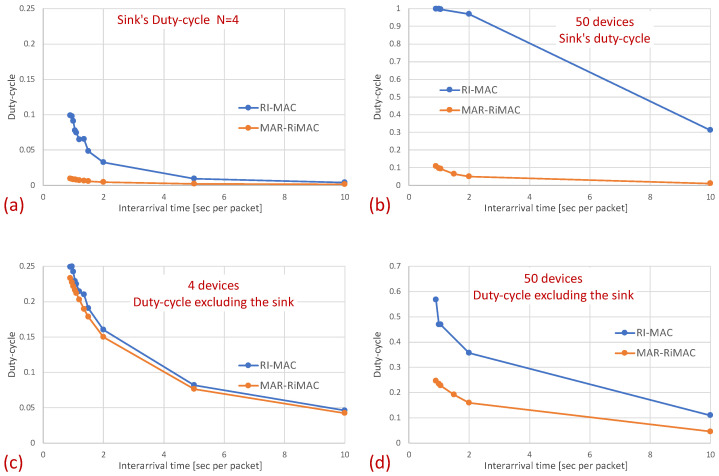
Hidden-terminal topology: (**a**) The sink’s mean duty cycle for four devices. (**b**) The sink’s mean duty cycle for 50 devices. (**c**) The mean duty cycle excluding the sink for four devices. (**d**) The mean duty cycle excluding the sink for 50 devices.

As can be seen in the figure, the sojourn time of RI-MAC is, as before, slightly shorter than that of MAR-RiMAC. However, this slightly shorter sojourn time is at the expense of a higher duty cycle, especially for high loads and for a moderate to high number of devices. The insight follows the same insight as with the clique topology, only the phenomenon is exaggerated. In particular, the collision probability, as well as the collision resolution time, for MAR-RiMAC is much lower than that of RI-MAC. Even for a low number of simultaneous-transmission devices supported by MAR-RiMAC (K=4), both the devices and the sink stay awake for much shorter time intervals to resolve collisions, and can go back to sleep much faster, i.e., the average duty cycles are lower. Consequently, since in RI-MAC, the sink stays awake for longer time intervals, more devices are waking up when the sink is awake; hence, they do not need to wait for the sink’s wake-up time before starting to transmit their reports, thus shortening their expected delay. Furthermore, since in RI-MAC, devices are awake for longer time intervals to resolve collisions, they receive more packets while both they and the sink are awake, and can transmit them on the same cycle, back to back with their packets that arrived earlier, again shortening their expected delay. We explore the aforementioned long awake interval of the sink by exploring its duty cycle (recall that Figure 11 depicts the average duty cycle of all the devices). Figure 12a,b depicts a comparison between the sink’s duty cycle for MAR-RiMAC and RI-MAC for the four- and 50-device topologies with hidden terminals. Figure 12c,d depicts the devices’ average duty cycle, excluding the sink. It can indeed be seen that, with high loads, the sink’s duty cycle in RI-MAC is much higher than the corresponding duty cycle in MAR-RiMAC. We further see that for the four-device topology, the sink’s duty cycle in MAR-RiMAC is lower than the devices’ average duty cycle (excluding the sink). However the sink’s low duty cycle confirms the observation that a large part of the devices’ awake time is spent waiting for the sink to wake up. Since in the presented results, the sink is never backlogged, it does not need to send data; hence, it does not need to wait for devices to wake up. After waking up and receiving all of the data (as there is no contention when K=4), it can go back to sleep. Note that even in the 50-device topology, despite the contention, the sink’s duty cycle is lower than the devices’ average duty cycle. In contrast, in RI-MAC, the sink is awake for long time intervals, allowing the backlogged devices to transmit to it. However, under these high loads, due to the high contention, the devices spend much more time resolving collisions.

**Figure 13:**
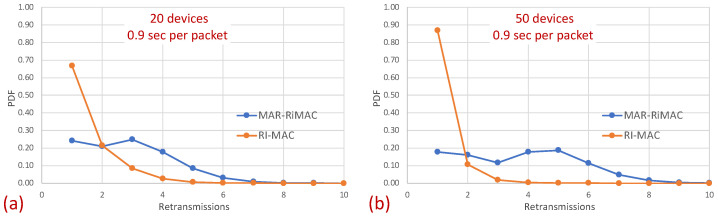
Hidden-terminal topology: The probability density distribution (PDF) of the number of retransmissions in RI-MAC. (**a**) Twenty devices. (**b**) Fifty devices.

The figures indeed confirm the supposition that, in RI-MAC, under heavy and moderate loads, the sink stays awake for longer time intervals. Note that for the 50-device topology, under a high load (inter-arrival times below 2 packet/s), the sink in RI-MAC is awake most of the time, while for MAR-RiMAC, even for the highest depicted load (0.9 packet/s, which is more than one packet per cycle per device), the sink is awake only slightly more than 10% of the time (11%). We further examine the protocol behavior under a high load, and for each successfully transmitted packet, we record how many unsuccessful tries (collisions) it has experienced. Specifically, Figure 13 depicts the probability density function (PDF) of the number of transmission attempts that each packet has experienced before being transmitted successfully for the 50-device topology and for an inter-arrival time of 0.9 packet/s. Recall that in MAR-RiMAC, each packet is transmitted only once when the sink polls the device for transmission, and in case of contention, the resolution is performed on the “Ready to Receive” (RtR) beacons, which are very short (i.e., their transmission time is significantly shorter than that of packet transmission). Accordingly, in the figure, we show the number of “Ready to Receive” (RtR) beacons sent before the sink polls the device for transmission.

**Figure 14:**
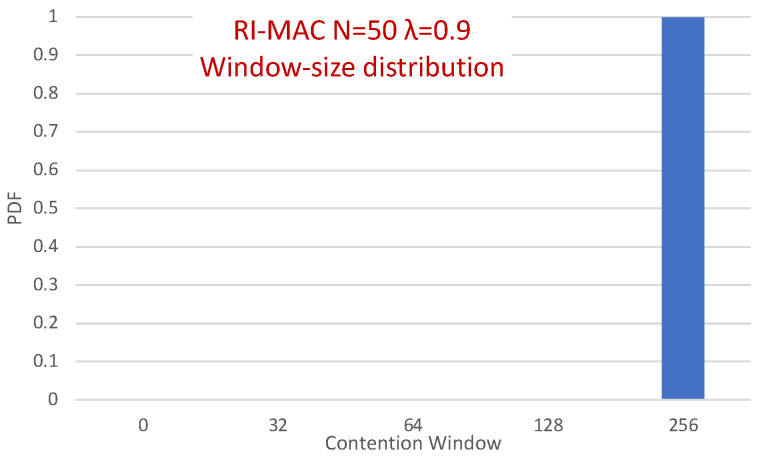
Hidden-terminal topology: RI-MAC contention window size distribution (PDF) for N=50 and λ=0.9.

Surprisingly, in Figure 13, it is evident that the packets in RI-MAC experience fewer transmission attempts than in MAR-RiMAC, which seems counterintuitive because of the fact that the duty cycle of the latter is much shorter for the depicted load. However, two important properties need be recalled. First, as previously mentioned, the transmission duration of RtR in MAR-RiMAC is much shorter than the packet transmission duration in RI-MAC. In addition, their transmission attempts are back to back with no gaps or contention intervals (recall that if more than *K* devices have attempted to transmit a reservation, the sink sends an RtR at the following slot, which immediately invites the colliding devices to randomly select whether to attempt again in the following slot). Consequently, the time duration required to resolve multiple collisions between the RtRs in MAR-RiMAC is significantly shorter than a packet transmission, all the more so when considering the contention intervals incurred by the RI-MAC contention mechanism. Second, in RI-MAC, after each collision, the sink increases the window size until successful transmission or up to the maximal contention window size (256 in the simulations, which provides good results). Since the sink cannot know how many backlogged devices there are at any time instance, the window is reset only when the sink goes to sleep. Accordingly, on each cycle, the window in RI-MAC is set to a size that balances between too short, resulting in repeated collisions, and too long, eliciting long idle intervals. Respectively, on each cycle, after several collisions, the contention window stabilizes on a large enough length that allows low collision probability at the expense of longer contention intervals (longer idle intervals). Accordingly, the sink is awake for long time intervals such that not all backlogged devices are transmitting their RtTs concurrently while they are waiting for the sink to wake up. Many of them wake up while the sink is already awake, and the announced window size in the sink’s RtR is sufficiently large to reduce the number of collisions. In order to validate this assertion and to explore the dynamics of RI-MAC contention window, we recorded the contention window announced by the sink for each successful packet. Figure 14 depicts the distribution of the contention window size with which each successful packet was transmitted under RI-MAC for 50 devices and λ=0.9.

The figure clearly depicts that for high loads, practically all packets were transmitted in the largest window (256). Recall that for this high traffic load, the sink was awake throughout the simulation duration (see Figure 12), which means that after waking up for the first time, the sink stayed awake throughout the simulations. Accordingly, due to the high load, after resolving the contention between many backlogged devices, the window was set at the appropriate window size for such a high load (256) and stayed at this size throughout the simulation duration. Note that, in the presence of hidden terminals (like the topology for which the results are presented in the figure), collision can occur not only upon transmission initiation, but also during the transmission itself (i.e., after a transmission has been started by a device, a hidden device that cannot sense the transmission can initiate its own transmission). However, since we adopted the parameters used in [9], in which the packet size is quite small (only 28 bytes), the packet transmission time is short; hence, the vulnerable period is also short, and the effect of collisions between hidden terminals in RI-MAC is not dramatic. Obviously, when enlarging the packet size, the effect on RI-MAC’s performance can be much more severe, while it affects the performance of MAR-RiMAC only in the transmission time and has no effect on the MAR-RiMAC contention mechanism.

**Figure 15:**
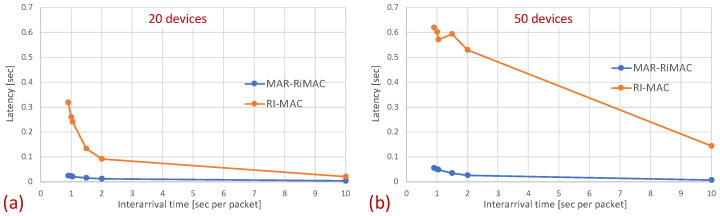
Hidden-terminal topology: The average latency experienced by the packets from the time that both the transmitting device and the sink are awake until the successful reception of the packet by the sink for: (**a**) 20 devices; (**b**) 50 devices.

As previously explained, the longer sojourn time experienced by MAR-RiMAC with respect to RI-MAC can be associated with the lower sink duty cycle. In particular, in MAR-RiMAC, since both the sink and the devices are awake for short time intervals, a device receiving a packet for transmission needs to first wait for its awake time and then until the sink’s awake time before attempting to transmit the packet. In contrast, in RI-MAC, since both the sink and the devices are awake for longer time intervals, it is more likely that when a device is receiving a packet, both the device and the sink are awake; hence, the longer waiting time for both to be awake can be skipped. In order to filter the waiting time for the device and sink to be awake and to evaluate the delay due to collisions, we measure the latency from the time epoch in which the packet is queued at the device and both the sink and the device are awake until the packet is transmitted successfully, i.e., if the backlogged device wakes up while the sink is asleep, we measure the time elapsed from the sink’s wake-up to the successful reception of the packet by the sink. If the backlogged device wakes up while the sink is awake, we measure the time elapsed from the device’s wake-up time to the successful reception of the packet by the sink. Figure 15 depicts the comparison of this latency between the two protocols for 20 and 50 devices.

It can be seen that when both the device and sink are awake, the average delays experienced by packets are much longer for RI-MAC than for MAR-RiMAC due to the longer collision resolution intervals, i.e., the latency of a packet because it is backlogged and both the device and the sink are awake is much shorter for MAR-RiMAC. Obviously, keeping the sink and devices awake all the time or for long time intervals (high duty cycle) will shorten the latency at the expense of energy consumption. This contradicts one of the basic goals of typical WSNs. Note that the packet formation instance is application-dependent. Hence, the time between packet formation and sink wake-up, which determines a lower bound on the sojourn time, cannot necessarily be coordinated. However, in both protocols, the device can synchronize additional awakening time with its intended receiver (the sink) such that a device will have its awakening time instance as a receiver, and in case it is backlogged, it will schedule an additional wake-up instance according to its intended receiver’s wake-up time (e.g., Wise-MAC [45] and PW-MAC [43]), which, in turn, will reduce the duty-cycle.

The results presented above are averaged over all devices that are dispersed over the inspected region; hence, they combine devices that are in sensing range of one another and others that are hidden from one another. One might suspect that the performance degradation of devices that have more hidden terminals is offset by the performance of devices with no or few hidden terminals. Accordingly, we examine the performance of one of the devices that is located in one of the corners. Note that the chosen device contends with devices that are hidden from it (dispersed around the other three corners of the region) and with devices that are within its sensing range.

**Figure 16:**
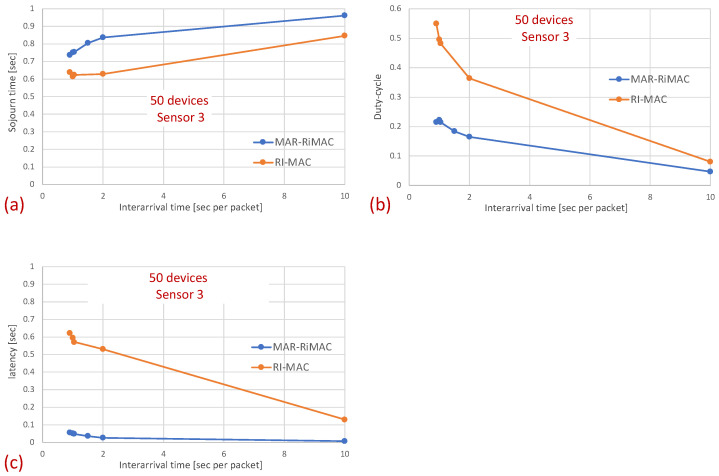
Hidden-terminal topology: The performance of a specific device (Device 3) for a topology comprising 50 devices under various mean inter-arrival times (λ). (**a**) Sojourn time. (**b**) Duty cycle. (**c**) Latency because both the transmitting device and the sink are awake.

Surprisingly, as can be seen in Figure 16, even though performance is expected to be more exposed to hidden devices and, hence, to suffer from more collisions than other devices, the results attained by the selected device (Device-3) in all three performance metrics (sojourn time, duty cycle, and latency because both the transmitting device and the sink are awake) are quite similar to the average ones. However, recall that, since the packet transmission time is quite short compared to the cycle itself, the penalty for collision is negligible. Moreover, each collision delays all queueing packets and keeps all backlogged devices awake regardless of whether they were the ones involved in the collision. All the more so, since the window grows for all the devices collectively, collisions by any devices, regardless of their location or topology, affect all the devices’ contention windows similarly; hence, their performance is equally affected.

**Figure 17:**
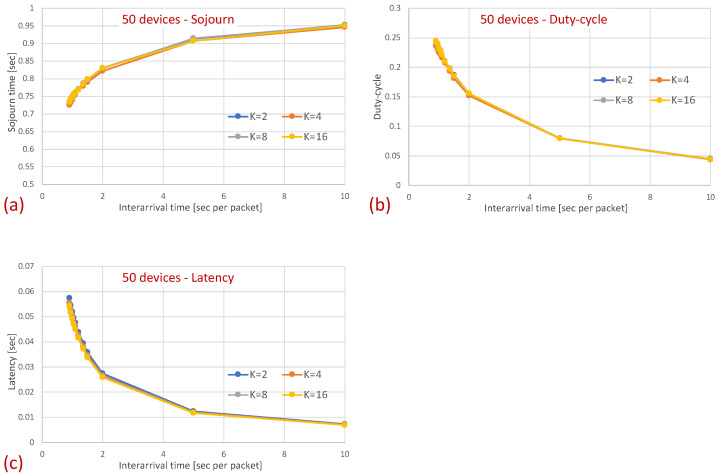
Hidden-terminal topology: The performance of a topology comprising 50 devices under various mean inter-arrival times (λ) and K=2,4,8,16. (**a**) Sojourn time. (**b**) Duty cycle. (**c**) Latency because both the transmitting device and the sink are awake.

Next, we examine the effect of *K* on the performance of MAR-RiMAC. Recall that *K* defines the number of reservations that can be transmitted simultaneously. We examined different *K*s (K=2,4,8,16) in the 50 devices with a hidden-device topology under different loads. Figure 17 shows the sojourn time, duty cycle, and latency.

Surprisingly, Figure 17 clearly depicts that *K* has a marginal effect on all three performance criteria. Since a greater *K* results in a higher number of reservations that can be transmitted and be decoded simultaneously, one could have expected that MAR-RiMAC performance would improve when increasing *K*, all the more so since, in our simulations, we kept a fixed *T*, i.e., the overhead while increasing *K* was kept constant. However, in MAR-RiMAC, even for small *K*, when more than *K* devices try to reserve a transmission simultaneously and a collision between these reservations will occur, not only is the collision very short (RtT) signals are very short), but the collision resolution procedure is also very short, and the loss due to this collision resolution mechanism is negligible compared to the cycle duration. Indeed, a sharp-eyed observer can notice that, for the high load end, the greater *K* is, the lower the latency, i.e., the latency when K=16 is slightly smaller than that of K=8, which is smaller than that of K=4, which is smaller than that of K=2, yet the differences are negligible (the difference between K=2 and K=4 is 3 ms; that between K=4 and K=8 is in the order of 1 ms; between K=8 and K=16, the difference is around 0.5 ms.

**Figure 18:**
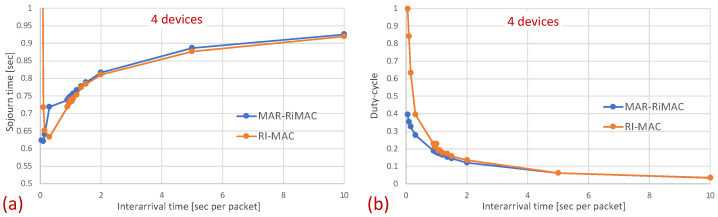
Hidden-terminal topology: The performance attained by a four-device topology under very heavy to light traffic loads. (**a**) Sojourn time. (**b**) Duty cycle.

Last, we examine a simple four-device topology, in which each device is hidden from the other three devices. We examine the setup under various traffic loads, starting from extremely heavy traffic (mean inter-arrival time 0.05 packet/s), which can represent a situation of an emergency event that requires the devices to send reports frequently (depicted in Figure 18).

Figure 18 depicts similar trends to those of the denser topology, in which, for high loads, the duty cycle of MAR-RiMAC is lower than that of RI-MAC at the expense of a slightly longer sojourn time. For light loads in which the sink can easily cope with the traffic, both the duty cycle and the sojourn time coalesce for both protocols. Note that, for very heavy traffic, MAR-RiMAC manages to cope with the intense load; hence, the devices manage to go to sleep. In contrast, RI-MAC cannot resolve collisions as fast as the arrival rate, i.e., the packet arrival rate is higher than the departure rate. Hence, all of the devices are awake at all times, and the sojourn time of RI-MAC is dramatically longer than the sojourn time of MAR-RiMAC. As soon as RI-MAC can resolve collisions at a faster rate than their arrival time, the sojourn time decreases dramatically, as does the duty cycle, as the devices can go to sleep. In this simulation section, we showed that the MAR-RiMAC amendment improves the performance and the air-time utilization in cases of high contention, when multiple devices are trying to communicate with the same receiver at the same time. It was shown that, in cases of sparse traffic, MAR-RiMAC preserves the performance attained by RI-MAC, despite the slight overhead. We believe that the dense network setup with high contention between devices—in which, even though each device has light traffic to transmit, the aggregated traffic from the numerous devices in the neighborhood causes high contention—is quite prevalent in current wireless sensors and IoT, and is going to be even more prevalent in future networks.

## 5. Discussion

In this paper, we devised an enhancement to receiver-initiated MAC protocols for duty-cycle-based wireless sensor networks. Our enhancement addresses the perpetual collisions for heavy traffic loads in which many devices are trying to transmit to the same entity (sink or relay). Such a collision storm is inherent in the conventional receiver-initiated MAC protocols for such a setup, as the receiver’s invitation to transmit a beacon initiates a transmission from multiple devices, resulting in a tedious contention resolution procedure.

Our enhancement, termed MAR-RiMAC, relies on a reservation-based mechanism in which the reservations are short signals that can be transmitted simultaneously. After the designated receiver decodes the identity of the multiple devices, it sends a transmission request and it polls them consecutively, one after the other, with no idle intervals. We implemented both MAR-RiMAC and RI-MAC (the original receiver-initiated MAC protocol) and thoroughly examined their behaviors under diverse traffic loads. We showed that, under heavy traffic loads, MAR-RiMAC can resolve contention very efficiently and much faster than RI-MAC at the expense of a small overhead of short signaling messages, which is negligible even under light loads.

## Figures and Tables

**Table 1:** Simulation radio parameters.

Bandwidth	250 Kbps	Size of Hardware Preamble	6 B
Preamble	192 μs	Size of ACK	5 B
Slot time	320 μs	CCA Check Delay	128 μs
